# Comparative kinematic analysis of forelimb and hindlimb cushioning strategies in German Shepherd dogs: implications for injury prevention

**DOI:** 10.3389/fvets.2026.1878605

**Published:** 2026-07-07

**Authors:** Huaibin Miao, Lin Zhu, Long Zheng, Zhihui Qian, Luquan Ren

**Affiliations:** 1 School of Mechanical and Electrical Engineering, Zhoukou Normal University, Zhoukou, China; 2 College of Energy and Power Engineering, Nanjing University of Aeronautics and Astronautics, Nanjing, China; 3 Key Laboratory of Bionic Engineering, Jilin University, Changchun, China

**Keywords:** canine biomechanics, forelimb–hindlimb differentiation, impact cushioning, injury prevention, sports medicine

## Abstract

**Objectives:**

While German Shepherds are widely utilized in agility and working roles, the limb-specific kinematic strategies they employ to attenuate impact during locomotion, and how these strategies relate to clinical injury susceptibilities, remain insufficiently understood.

**Methods:**

In this preliminary study, we used a three-dimensional motion capture system to quantify the forelimb and hindlimb kinematics of four clinically healthy German Shepherd dogs during walking, trotting, and controlled drop-landing.

**Results:**

Joint angle profiles revealed significant functional differentiation: forelimbs exhibited inverted pendulum-like kinematics prioritizing initial contact stability, whereas hindlimbs demonstrated spring-like kinematics specialized for propulsion. Crucially, we identified distinct limb-specific cushioning mechanisms during initial ground contact: forelimbs passively absorb impact via rapid carpal hyperextension (dorsiflexion), while hindlimb tarsal flexion geometrically implies active eccentric muscular control.

**Conclusion and relevance:**

These findings provide essential preliminary kinematic baseline data for healthy German Shepherds. The passive forelimb strategy is biomechanically consistent with the high clinical incidence of carpal ligamentous injuries, whereas the active hindlimb strategy may predispose the stifle to fatigue-related cranial cruciate ligament insufficiency. These insights deepen our understanding of functional limb differentiation and establish a critical normative reference for evaluating pathological gait deviations, informing targeted conditioning programs in veterinary sports medicine and orthopedic rehabilitation.

## 1 Introduction

German Shepherds are widely utilized in agility (1), police (2), and military (3) roles, subjecting their musculoskeletal systems to high-velocity and high-impact locomotor demands. Consequently, these working dogs are highly susceptible to orthopedic injuries, with front limb carpal sprains and hind limb cranial cruciate ligament (CCL) ruptures being among the most prevalent and career-ending conditions (4–6). Understanding the underlying biomechanical mechanisms that predispose these specific joints to injury is crucial for developing targeted conditioning and prophylactic strategies. Recent systematic reviews have established baseline kinematic parameters for healthy dogs (7), and comprehensive reviews have further detailed recent methodological developments in canine locomotor analysis (8), yet detailed kinematic analyses during high-impact landing remain underrepresented.

Quadrupedal locomotion is traditionally described through the lens of energy-saving mechanisms, specifically the inverted pendulum model—which exchanges kinetic and potential energy during walking—and the spring-loaded inverted pendulum model, which utilizes elastic strain energy in tendons to maximize efficiency during running and trotting (9, 10). Within this framework, the forelimbs and hindlimbs exhibit distinct functional specializations (11, 12). The forelimbs primarily provide braking and gravitational support during initial contact, whereas the hindlimbs are dedicated to forward propulsion (13, 14). This functional dichotomy is evident in ground reaction force distributions, where the forelimbs bear a disproportionately larger share of the vertical load (15). Recent comparative studies have further elucidated breed-specific biomechanics; for instance, Humphries et al. (16) highlighted distinct kinetic and kinematic patterns between German Shepherds and Labrador Retrievers during standing and trotting. Furthermore, inherent variability in canine gait analysis, as emphasized by Holler et al. (17), necessitates precise methodological controls when evaluating these functional differences.

However, energy dissipation during high-speed or jump-landing impacts is arguably as critical as energy conservation for preventing tissue overload. Mammals employ various mechanisms to attenuate impact shocks, relying heavily on passive structures such as tendons and ligaments, which act as dampers to absorb energy and protect joints (18, 19). Furthermore, the morphology and material properties of the footpads provide a crucial first line of defense against ground impacts, functioning as a nonlinear, viscoelastic, multi-layered cushioning system (20–24). While these phenomena have been studied in feline landing mechanics (25, 26), the specific limb-level kinematic strategies utilized by canines to mitigate landing impacts—particularly the balance between active muscular engagement and passive ligamentous stretching across the limbs—remain insufficiently explored (27, 28). In German Shepherds specifically, conformational traits such as hindlimb angulation significantly alter kinematic patterns, which may predispose them to specific orthopedic conditions (29). Recent quantitative studies on agility dogs have begun to characterize jump landing patterns, emphasizing both universal biomechanical patterns and individual technique variability (30), including studies that combine kinetic and kinematic assessments (31).

We hypothesized that the forelimbs and hindlimbs adopt distinct kinematic strategies during impact absorption, and that these strategies are biomechanically plausible in the context of the clinical predispositions to specific orthopedic injuries observed in this breed. Therefore, the objective of this study was to quantify the three-dimensional limb kinematics of German Shepherd dogs during walking, trotting, and controlled drop-landing, aiming to identify limb-specific impact-cushioning mechanisms and to interpret these patterns in light of common orthopedic injuries in German Shepherds.

## 2 Materials and methods

### 2.1 Experimental subjects

Four clinically healthy adult male German Shepherd dogs from the Changchun Police Dog Base were selected for this study. The dogs had a mean age of 4.0 ± 0.5 years, a mean body mass of 33.0 ± 3.7 kg. Prior to data collection, all dogs were subjected to a thorough orthopedic examination by a licensed veterinarian to confirm the absence of any musculoskeletal disorders or lameness that could affect their natural gait. The experimental protocol was reviewed and approved by the Institutional Review Board of Jilin University (Approval No. 20140418), and all procedures strictly complied with the relevant guidelines for the care and use of animals.

### 2.2 Motion capture and experimental protocol

Three-dimensional limb kinematics were recorded using an eight-camera infrared motion analysis system (VICON MX, Vicon Motion Systems, Oxford, UK) operating at a sampling rate of 125 Hz. A custom marker set comprising 27 reflective markers (8 mm diameter) was applied to anatomical landmarks to define segment kinematics. Markers were placed bilaterally on the thoracic and lumbar vertebrae, bony landmarks of the scapula and pelvis, and on the shoulder, elbow, carpus, third metacarpophalangeal joint, and the distal end of the third digit of the forelimbs. For the hindlimbs, markers were placed on the hip, stifle, tarsus, third metatarsophalangeal joint, and the distal end of the third digit. To minimize discomfort and maintain the practical relevance for working dogs, markers were securely attached to the fur using double-sided adhesive tape without shaving the coat (32). Before data collection, the system was statically and dynamically calibrated, yielding a mean residual camera measurement error of 0.07 mm (32).

All dogs were assessed during three locomotor conditions: walking, trotting, and a controlled drop-landing task. The drop-landing task involved the dogs dropping off a standardized 0.5-m elevated platform, which simulates the descent from agility obstacles. Walk and trot trials were primarily distinguished by their foot-ground contact patterns (33). Specifically, walking was identified by a three-limb support pattern (three legs simultaneously in contact with the ground), whereas trotting was identified by a diagonal bipedal support pattern (diagonal fore- and hindlimbs simultaneously in contact with the ground). Walking and trotting were included as low and moderate impact baseline conditions, respectively, to contextualize the high impact kinematic strategies observed during the drop-landing task. For each dog, 10 valid trials were collected per locomotor condition. Marker trajectories were automatically labeled and reconstructed using Vicon Workstation software (Vicon Motion Systems, UK). Occasional missing data points (typically less than 5 frames) were corrected via spline interpolation based on adjacent marker trajectories. Reconstructed data were exported as C3D files for further processing.

Kinematic parameters, including joint angles and segment displacements were extracted using the Polygon Authoring Tool (Vicon Motion Systems, UK), which was also utilized to generate stick figure diagrams representing the three locomotor conditions. Given that the drop-landing task constituted a non-cyclic movement rather than a steady-state gait, full gait cycles were not analyzed; therefore, the drop-landing data were exclusively utilized to investigate limb impact-cushioning strategies during the stance phase.

### 2.3 Statistical analysis

Kinematic data were compiled and pre-processed using Microsoft Excel (Microsoft Corp., United States). Unless stated otherwise, all data are expressed as the mean ± standard deviation (SD). Statistical analyses were performed using SPSS 22.0 (SPSS Inc., Chicago, IL, United States). The Shapiro–Wilk test was used to verify the normality of the data distribution. The individual dog was the experimental and statistical unit (n = 4). For each condition, the 10 valid trials were averaged within each dog, and these four dog-level means were used for all subsequent statistical analyses. The individual dog-level means for kinematic variables (joint angles and displacements) are provided in Supplementary Table S1. Because all three locomotor conditions (walking, trotting, and drop-landing) were performed by the same group of dogs, a one-way repeated-measures analysis of variance (ANOVA) was used to evaluate differences among the three conditions. For direct comparisons between the forelimbs and hindlimbs within a specific condition, paired-samples t-tests were applied. A *p* value of < 0.05 was considered statistically significant.

## 3 Results

### 3.1 Kinematic characteristics of the forelimbs and hindlimbs

**Figure 1:**
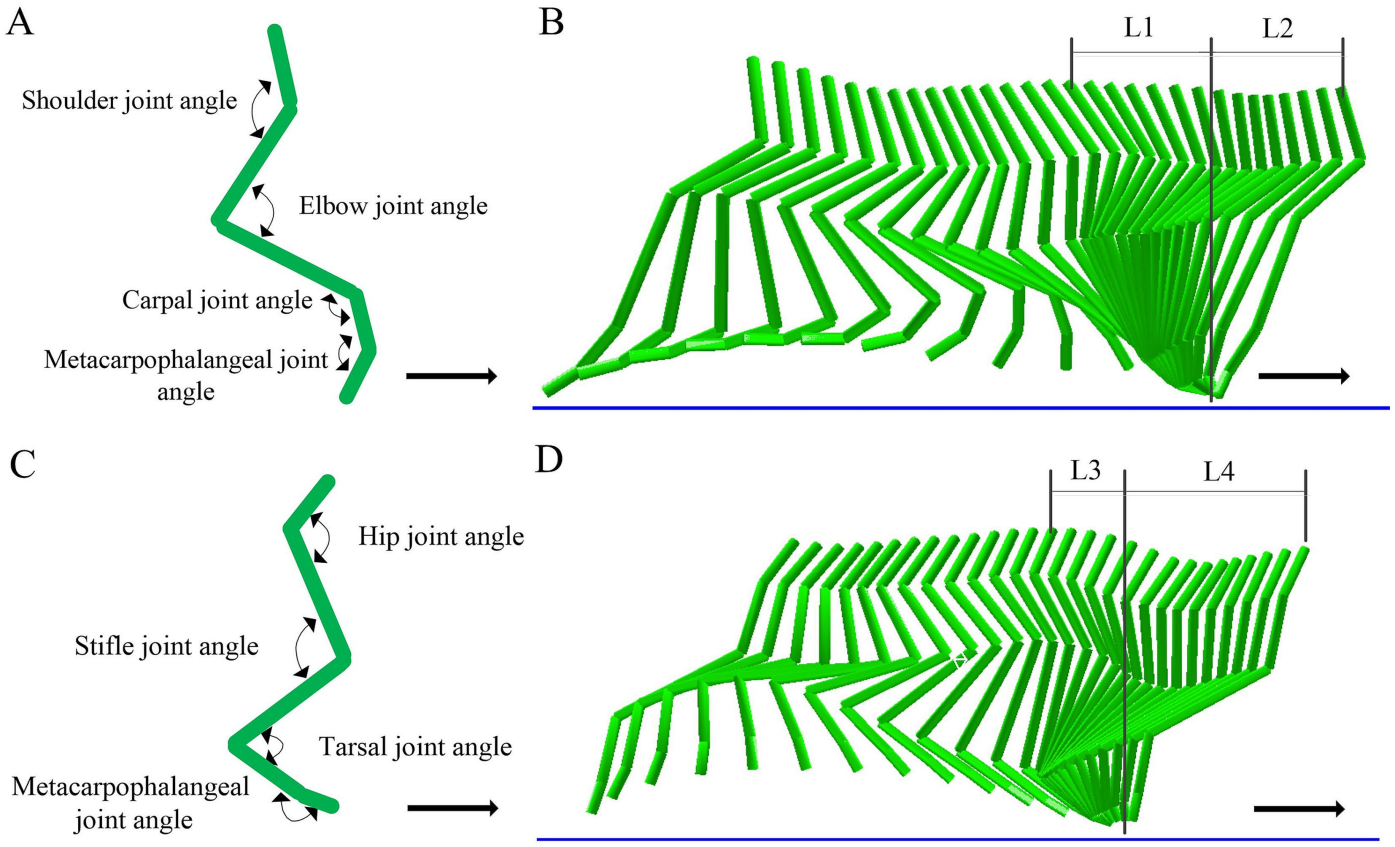
Joint angle definitions and kinematic trajectories of the forelimbs and hindlimbs in a German Shepherd during a trotting cycle. Arrows indicate the direction of forward progression. **(A)** Schematic definition of forelimb joint angles; **(B)** Forelimb spatial trajectory during a complete stride; **(C)** Schematic definition of hindlimb joint angles; **(D)** Hindlimb spatial trajectory during a complete stride. Three vertical black lines delineate the stance phase: the central longer vertical black line indicates the foot-ground contact point and serves as the spatial baseline, while the two shorter flanking vertical black lines mark the onset and offset of the stance phase, respectively. The horizontal distances between these lines define the braking and propulsion lengths: L_1_ (forelimb) and L_3_ (hindlimb) represent the horizontal distance from the left short vertical line to the central longer vertical line (braking phase); L_2_ (forelimb) and L_4_ (hindlimb) represent the horizontal distance from the central longer vertical line to the right short vertical line (propulsion phase).

In this study, joint angles were defined based on the active flexion directions of each joint. The anatomical definitions for the forelimb joints (shoulder, elbow, carpal, and metacarpophalangeal) and hindlimb joints (hip, stifle, tarsal, and metatarsophalangeal) are illustrated in Figures 1A,C, respectively. For the purpose of this analysis, an angle exceeding 180° was defined as joint hyperextension (dorsiflexion). Consistent with previous findings (34), the bilateral forelimb and hindlimb movements of the German Shepherds were symmetrical and highly periodic. Consequently, the present analysis focused exclusively on the kinematic data of the left forelimb and right hindlimb during a single movement cycle. While general kinematic patterns during walking and trotting align with established canine gait benchmarks, they are presented here as baseline references to contrast with the high-impact condition.

**Figure 2:**
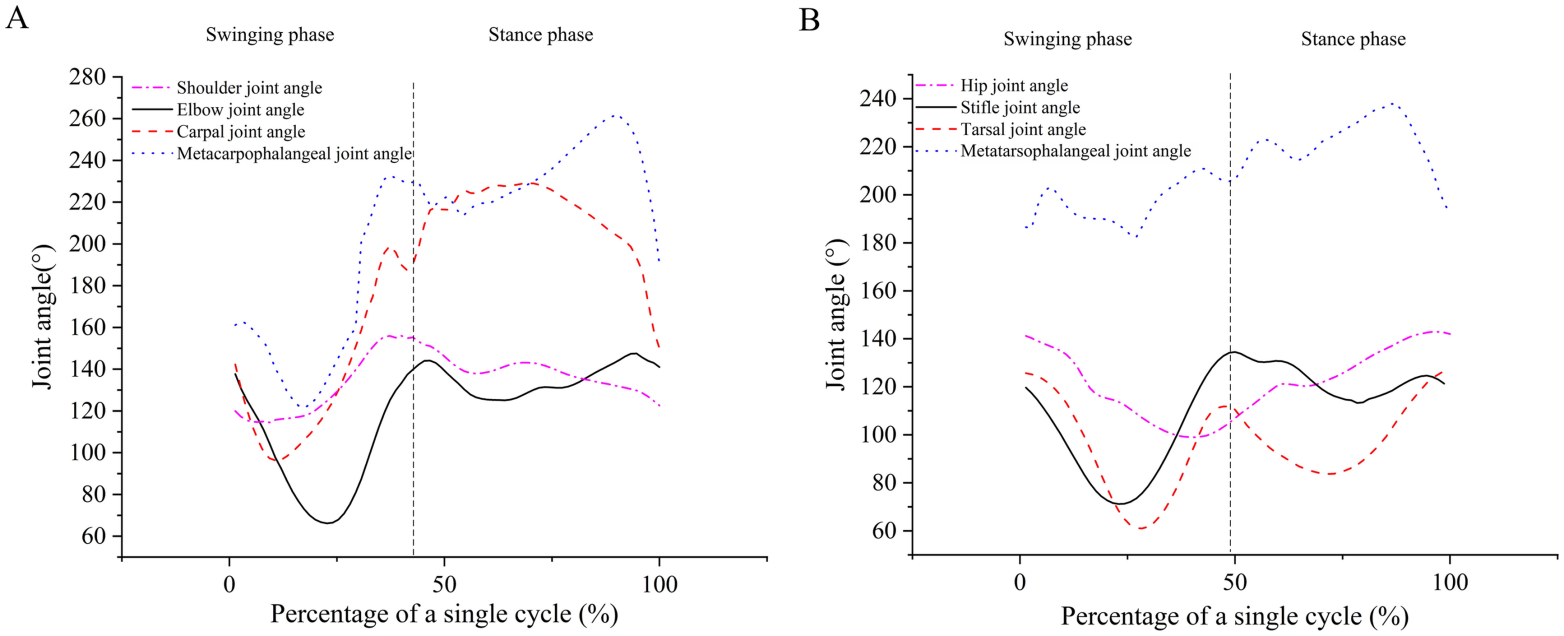
Variation patterns of joint angles in the forelimbs and hindlimbs of German Shepherds during trotting. **(A)** Forelimb joint angles; **(B)** Hindlimb joint angles.

A complete stride cycle for both the forelimbs and hindlimbs consists of a swing phase and a stance phase (Figures 1B,D). Visual inspection of the movement trajectories revealed markedly different locomotor patterns between the forelimbs and hindlimbs. Therefore, a detailed comparative analysis was conducted regarding the angular variations in the shoulder, elbow, carpal, and metacarpophalangeal joints of the forelimb (Figure 2A), as well as the hip, stifle, tarsal, and metatarsophalangeal joints of the hindlimb (Figure 2B).

Because the joint angle variation patterns during walking and trotting were highly consistent—differing only in the amplitude of angular changes—the following quantitative analysis focuses exclusively on the trotting gait. During trotting, the swing phase accounted for approximately 41% of the forelimb cycle and 49% of the hindlimb cycle (Figure 2).

During the swing phase, the forelimb shoulder joint primarily underwent extension, reaching its maximum extension angle immediately prior to touchdown, followed by flexion during the stance phase. The angular range of motion (ROM) was 38.2 ± 4.3° during the swing phase and 24.6 ± 3.6° during the stance phase (Figure 2A). In contrast, the hindlimb hip joint primarily flexed during the swing phase (reaching a minimum angle prior to touchdown) and extended during the stance phase. Its ROM was 45.3 ± 5.3° (swing) and 43.9 ± 5.1° (stance) (Figure 2B). Thus, the shoulder and hip joints exhibited reciprocal angular trends. While their swing phase ROMs were similar, the hip joint exhibited a substantially larger ROM during the stance phase.

The forelimb elbow joint exhibited a biphasic flexion-extension pattern during the swing phase, followed by slight continued extension and subsequent flexion during the stance phase. The ROM was 56.0 ± 7.3° (swing) and 29.7 ± 5.1° (stance) (Figure 2A). The hindlimb stifle joint demonstrated a similar rapid flexion-extension sequence in the swing phase, and gradual flexion in the stance phase, with an ROM of 63.5 ± 6.3° (swing) and 27.9 ± 4.6° (stance) (Figure 2B). The angular trajectories and amplitudes of the elbow and stifle joints were highly congruent.

The forelimb carpal joint also flexed and then extended during the swing phase. Prior to touchdown, the extension angle exceeded 180°, entering a hyperextended state. This hyperextension was maintained throughout almost the entire stance phase, resolving only toward the very end. The ROM was 88.7 ± 6.4° (swing) and 29.4 ± 4.2° (stance) (Figure 2A). The hindlimb tarsal joint executed a similar flexion-extension sequence during both the swing and stance phases, with an ROM of 56.5 ± 4.7° (swing) and 43.3 ± 6.1° (stance) (Figure 2B). Notably, the tarsal joint never exceeded 180°. The forelimb carpal joint exhibited a larger swing-phase ROM but a smaller stance-phase ROM compared to the tarsal joint, indicating distinct functional roles.

The forelimb metacarpophalangeal joint mirrored the carpal joint, maintaining a hyperextended state (angle > 180°) throughout the stance phase, with an ROM of 104.8 ± 9.7° (swing) and 38.8 ± 5.9° (stance) (Figure 2A). Conversely, the hindlimb metatarsophalangeal joint displayed no distinct pattern during the swing phase, but hyperextended and then extended during the stance phase, with an ROM of 40.6 ± 7.3° (swing) and 55.0 ± 8.6° (stance) (Figure 2B).

The stance phase trajectory of the forelimb was divided into a braking phase (L_1_, from initial contact to when the digit aligns vertically with the scapula) and a propulsion phase (L_2_, from vertical alignment to toe-off) (Figure 1B). The ratio of L_2_ to L_1_ was 1.05 ± 0.05, indicating near-symmetrical braking and propulsion. This supports the characterization of the forelimb as functioning similarly to an inverted pendulum, consistent with the spring-loaded inverted pendulum model (8). For the hindlimb, the trajectory was divided similarly relative to the pelvis (L_3_ and L_4_) (Figure 1D). However, the ratio of L_4_ to L_3_ was 2.3 ± 0.25, demonstrating a markedly longer propulsion phase, which indicates a dominant forward propulsion function for the hindlimb.

### 3.2 Ground contact cushioning strategies of the forelimbs and hindlimbs

**Figure 3:**
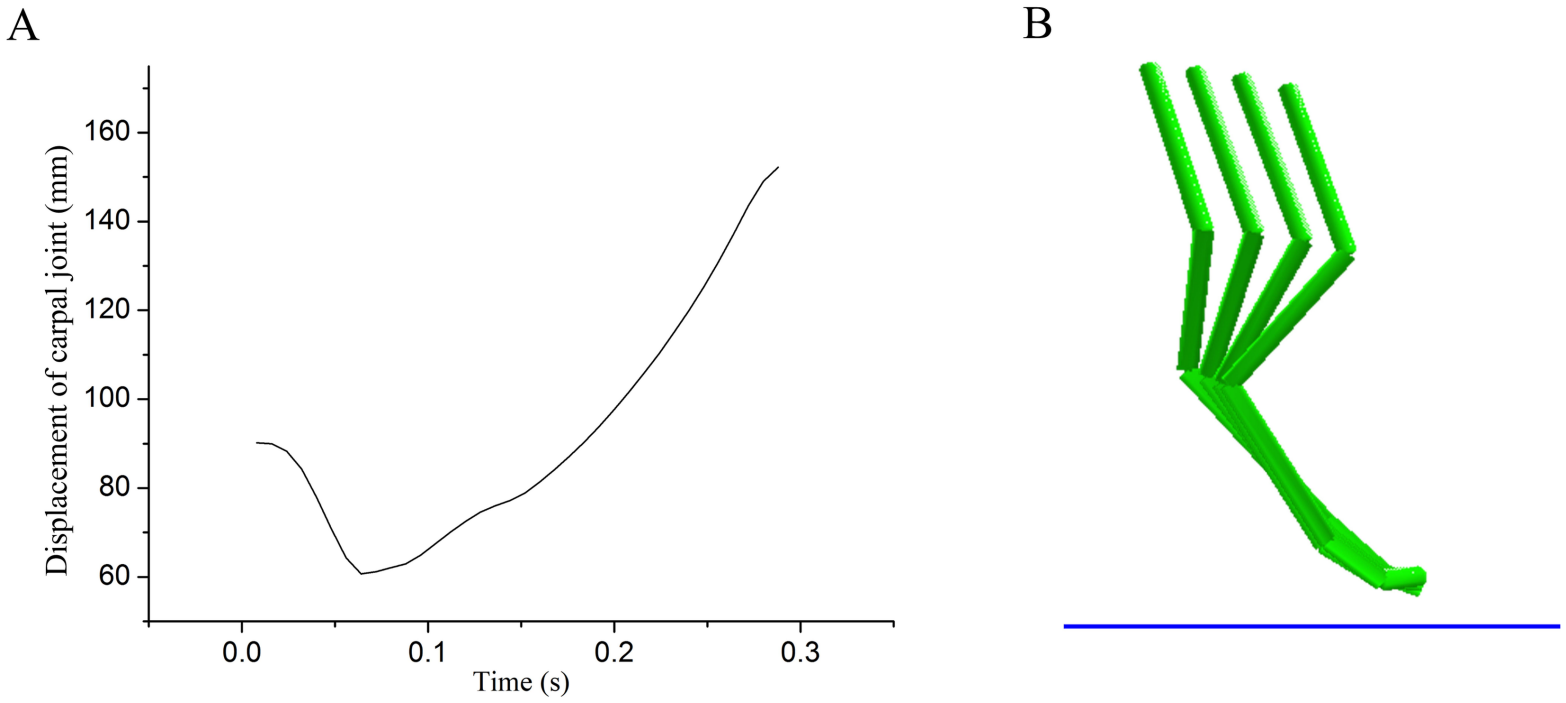
Vertical displacement of the carpal joint and trajectory of the forelimb during the ground contact phase. **(A)** Vertical displacement of the carpal joint; **(B)** Forelimb trajectory at the point of minimum vertical displacement.

Including walking, trotting, and the drop-landing task allowed us to observe a continuum of impact attenuation. Walking represents a low-impact baseline where passive structures are minimally loaded. Trotting introduces moderate elastic energy storage, while drop-landing represents the extreme high-impact scenario where the structural limits of the cushioning mechanisms are most evidently challenged. Analysis of the forelimb trajectory during initial contact revealed that the carpal joint rapidly moved downward, reaching its lowest vertical position within a short timeframe, before gradually ascending (Figure 3A). This rapid downward displacement coincided with a sharp increase in the carpal joint angle (hyperextension) (Figure 3B). Therefore, this initial rapid downward displacement phase of the carpal joint was defined as the landing cushioning phase for the forelimb.

**Figure 4:**
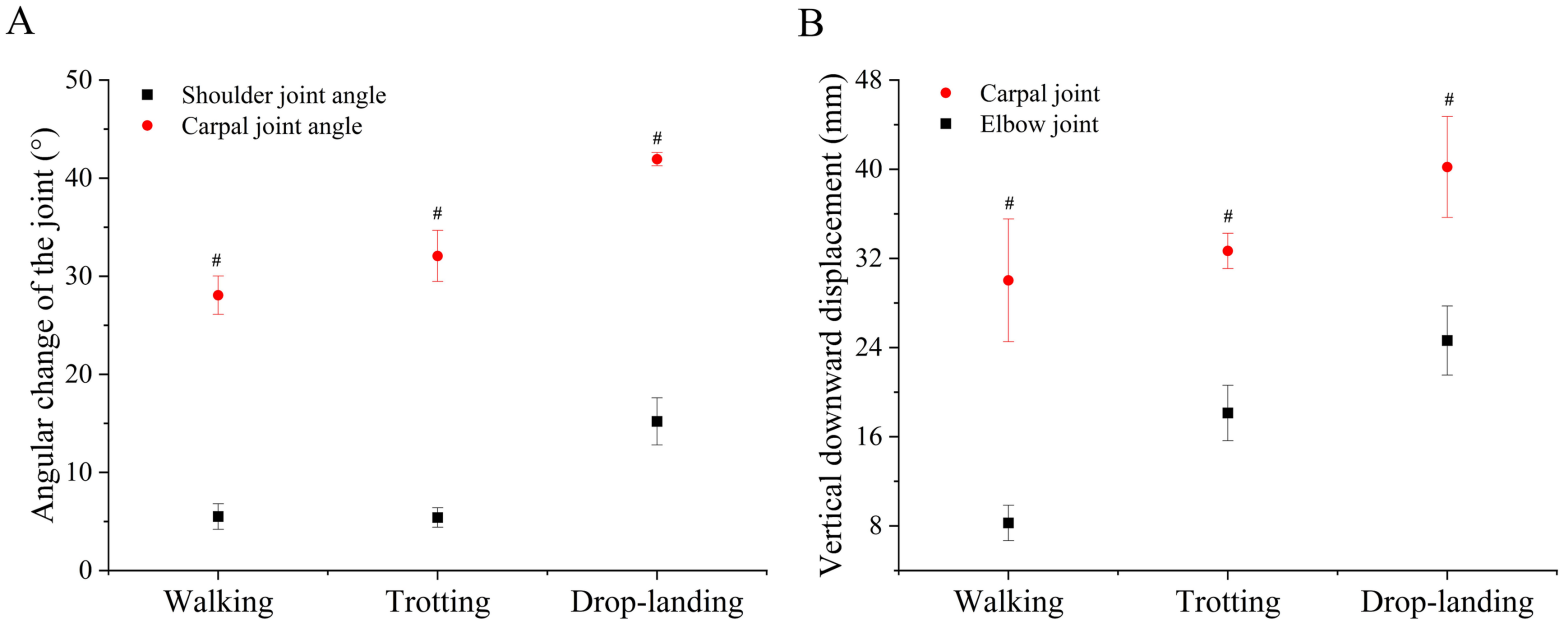
Joint angle variations and vertical displacements under three gait conditions. **(A)** ROM of the shoulder and carpal joints during the cushioning phase; **(B)** Vertical displacement ROM of the elbow and carpal joints during the cushioning phase. Data are expressed as the mean ± SD. # indicates a significant difference between the two joints within the same gait (*p* < 0.05).

Based on the forelimb angular profiles (Figure 2), during this cushioning phase, the shoulder joint flexed, the elbow joint extended and then flexed, the carpal joint underwent rapid hyperextension (>180°), and the metacarpophalangeal joint fluctuated irregularly. Across all three locomotor conditions (walking, trotting, and drop-landing), the angular change of the carpal joint during cushioning was significantly greater than that of the shoulder joint (*p* < 0.05) (Figure 4A). Furthermore, the vertical downward displacement of the carpal joint was significantly greater than that of the elbow joint in all conditions (*p* < 0.05) (Figure 4B) (individual dog-level data for these variables are provided in Supplementary Table S1). Given the distal location of the carpal joint, the data indicate that its rapid hyperextension is driven by the downward momentum of the distal limb segment, which kinematically engages the associated musculature and ligaments to absorb impact energy (35).

**Figure 5:**
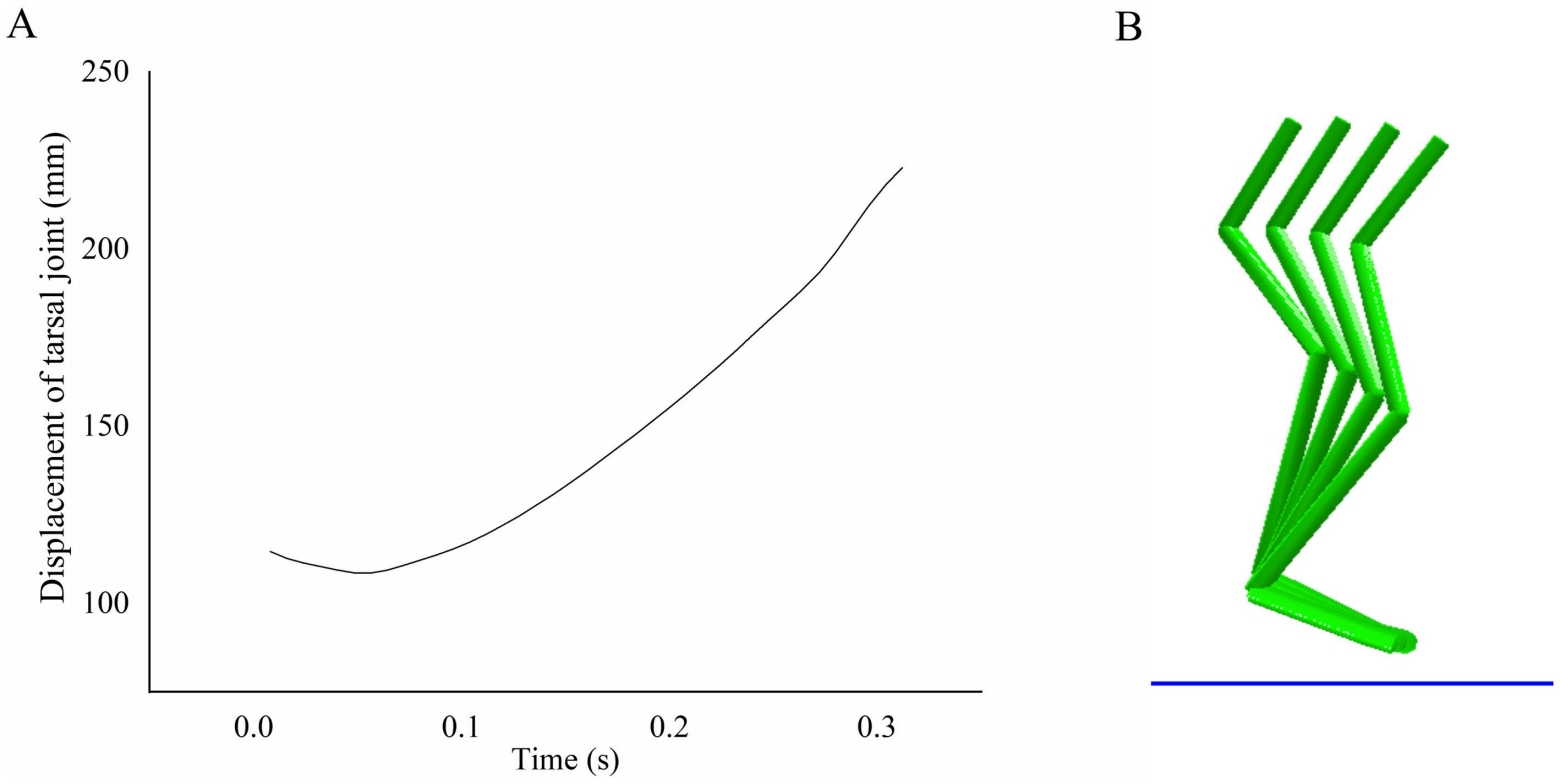
Vertical displacement of the tarsal joint and trajectory of the hindlimb during the ground contact phase. **(A)** Vertical displacement of the tarsal joint; **(B)** Hindlimb trajectory at the point of minimum vertical displacement.

Similarly, in the hindlimb, the tarsal joint rapidly moved downward to its lowest position immediately upon ground contact (Figure 5A), accompanied by distinct tarsal flexion (Figure 5B). Thus, the hindlimb cushioning phase was defined as the period from initial hindlimb contact to the point of maximum tarsal vertical displacement. During this phase, the hip joint extended while the stifle and tarsal joints flexed (Figure 2B), suggesting that impact attenuation is predominantly managed by the stifle and tarsal joints.

**Figure 6:**
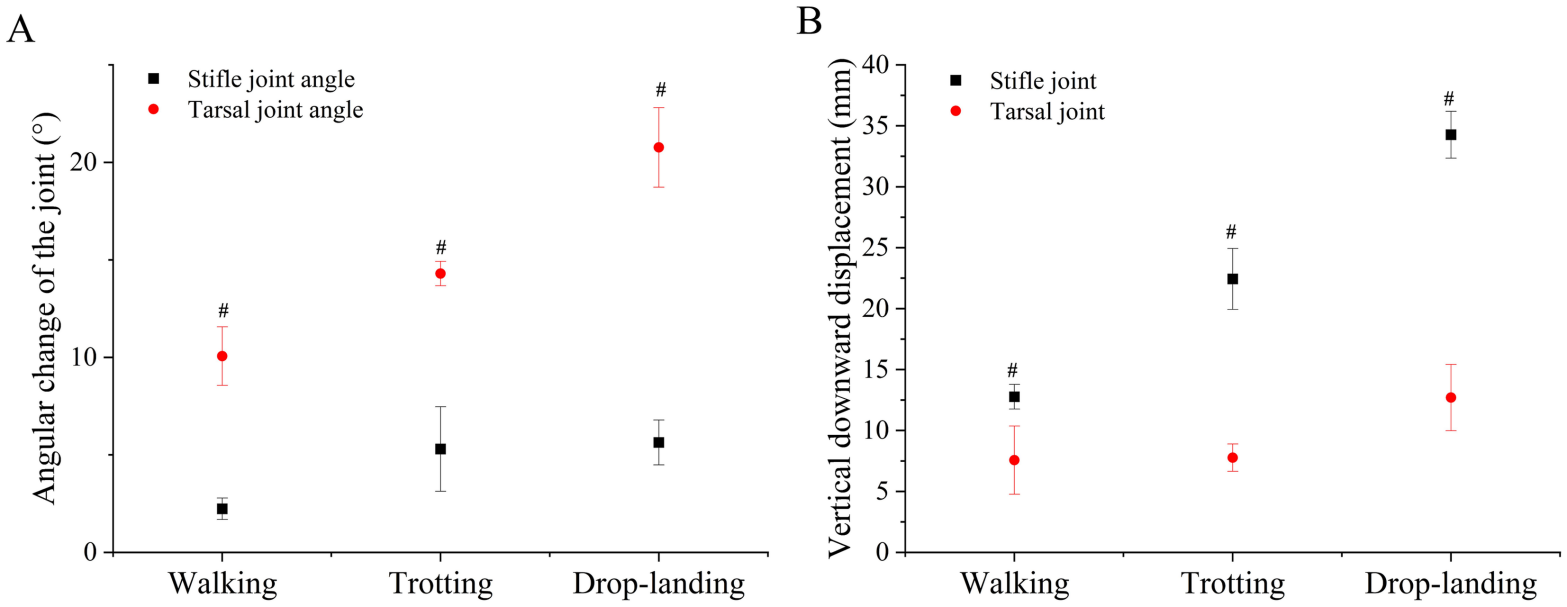
Joint angle variations and vertical displacements under three gait conditions. **(A)** ROM of the stifle and tarsal joints during the cushioning phase; **(B)** Vertical displacement ROM of the stifle and tarsal joints during the cushioning phase. Data are expressed as the mean ± SD. # indicates a significant difference between the two joints within the same gait (*p* < 0.05).

Across the three conditions, the flexion ROM of the tarsal joint during the cushioning phase was significantly greater than that of the stifle joint (*p* < 0.05) (Figure 6A). Interestingly, the vertical displacement of the stifle joint was significantly greater than that of the tarsal joint (*p* < 0.05) (Figure 6B). This indicates that tarsal flexion is mechanically driven by the greater downward displacement of the proximal segment (stifle). This kinematic pattern functionally resembles spring compression, effectively absorbing impact energy during landing.

## 4 Discussion

The present study conducted a comprehensive three-dimensional kinematic analysis of German Shepherds across walking, trotting, and controlled drop-landing. Our findings reveal a profound functional dichotomy between the forelimbs and hindlimbs, challenging the assumption of mechanical uniformity in quadrupedal gait. Most notably, we identified distinct, limb-specific cushioning strategies—an active muscular strategy in the hindlimbs versus a passive musculotendinous strategy in the forelimbs—that are biomechanically consistent with the clinical predisposition to specific orthopedic injuries in working and sporting dogs.

Consistent with previous force plate studies (27, 36), our kinematic data corroborate that the forelimbs and hindlimbs serve fundamentally different roles (9). During the swing phase, the forelimbs exhibited cranial trajectory arcs consistent with an inverted pendulum model, optimizing forward progression and potential energy conversion (9, 10). Conversely, the hindlimbs demonstrated caudal trajectories indicative of a compression spring mechanism, primed for propulsion and energy storage (13, 18). This divergence is critically amplified during the stance phase. Upon ground contact—particularly during the high-impact drop-landing task—the hindlimbs exhibited immediate and substantial flexion in the hip and stifle joints, characteristic of an active shock-absorbing mechanism. This observation aligns with Miqueleto et al. (29), who noted distinct pelvic limb kinematics in German Shepherds that are heavily influenced by breed-specific conformation. In stark contrast, the forelimbs maintained a relatively rigid proximal axis, with impact attenuation achieved primarily through large, rapid passive dorsiflexion of the carpal joint.

It is crucial to contextualize these limb-specific strategies within the breed-specific conformation of German Shepherds. Unlike other medium-to-large working breeds (e.g., Labrador Retrievers) that exhibit more extended hindlimb postures (15), German Shepherds possess a characteristic steep pelvic slope and excessive hindlimb angulation. This aberrant posture inherently pre-positions the stifle and tarsal joints in a relatively flexed state, which geometrically facilitates the “active cushioning” mechanism upon impact but simultaneously demands greater eccentric muscle control to prevent collapse. As Miqueleto et al. (29) demonstrated, even subtle changes in this angulation significantly alter pelvic limb kinematics. Therefore, the active shock-absorbing pattern observed in our study is likely exacerbated by GSD-specific conformation, which may predispose them to CCL injuries more readily than breeds with straighter hindlimb postures.

The kinematic pattern observed in the forelimbs defines what we term a “passive cushioning strategy.” Lacking the substantial eccentric muscle bulk of the hindlimb proximal joints, the canine forelimb relies on the rapid, gravity-driven extension of the elbow and extreme passive dorsiflexion of the carpus to dissipate impact energy. This mechanism kinematically subjects the palmar ligaments, joint capsule, and flexor tendons of the carpus to immense tensile loads almost instantaneously. *In-vivo* fluoroscopic studies have confirmed the extreme range of passive dorsiflexion the carpus undergoes during loading (37). Biomechanically, repetitive sub-maximal loading of passive collagenous structures leads to microtrauma, cumulative fatigue, and eventual structural failure (38). This provides a robust mechanistic explanation for the high clinical incidence of carpal hyperextension injuries, palmar ligament desmitis, and sprains reported in agility and working dogs (1, 4). Recent work on agility dogs has further highlighted the substantial impact forces and rapid joint angular excursions during jump landing, which likely exacerbate these passive loading conditions (30, 31). Additionally, recent clinical biomechanical insights emphasize that the unique anatomical constraints of the canine carpus render it highly vulnerable to acute and repetitive stress injuries during athletic activities (39). While the inherent cranial weight distribution of quadrupeds dictates that the forelimbs must absorb a disproportionate share of vertical ground reaction forces (15, 40), our data reveal that it is the passive nature of how these forces are absorbed—not merely their magnitude—that renders the distal forelimb inherently vulnerable. In working scenarios, external loads and leash forces further alter limb loading and may amplify the risk of carpal overload during landing and sudden deceleration (41).

In contrast, the hindlimb kinematic pattern is consistent with an “active cushioning strategy,” which geometrically implies substantial eccentric muscle control to decelerate the center of mass, although direct EMG confirmation is lacking. This active flexion of the hip and stifle decelerates the body’s center of mass smoothly, effectively dissipating kinetic energy as heat while protecting the joint capsules from acute tensile overload (19). However, this strategy carries a distinct metabolic and structural cost. High-intensity eccentric contractions are highly demanding on muscle fibers and place significant tensile stress on the tendons that restrain joint flexion—most notably the CCL at the stifle (5). Consequently, while the active hindlimb strategy is highly effective for both impact attenuation and subsequent propulsion, it may predispose the stifle to fatigue-related microinstability and catastrophic CCL rupture under conditions of exhaustion or overexertion (5). This trade-off aligns with recent biomechanical and clinical reviews emphasizing eccentric overload and repetitive microtrauma as central to CCLD pathogenesis (6, 42). While these kinematic patterns are biomechanically consistent with the known clinical predispositions, establishing direct causality would require longitudinal studies with clinical outcome data.

The limb-specific cushioning strategies identified in this study represent the second line of defense against ground impact, operating proximal to the footpads. Recent investigations into the material properties of the canine footpad reveal that it functions not as a simple linear spring, but as a highly sophisticated nonlinear viscoelastic structure (21, 22). Specifically, its morphological design allows for progressive stiffening under increasing loads—exhibiting high compliance during low-impact activities (e.g., walking) to smooth subtle vibrations, while rapidly increasing stiffness to dissipate massive kinetic energy during high-impact events (e.g., drop-landing) (20, 22–24). However, even with this nonlinear shock absorption, the kinetic energy during extreme drop-landing events inevitably exceeds the structural limits of the pad’s viscous damping. The transition from footpad compliance to joint-level cushioning highlights a synergistic cascading system: the footpads dampen the initial impact spike and buy critical milliseconds of time, forcing the proximal musculoskeletal system to engage limb-specific damping mechanisms. In the hindlimbs, this triggers the muscular pre-activation required for eccentric control; in the forelimbs, it permits the passive carpal mechanism to unfurl. When the pad’s nonlinear limit is breached, the residual unattenuated energy is transferred directly to the joints, thereby activating the passive carpal stretch that underlies the injury mechanisms discussed above (37).

Despite these contributions, certain limitations must be acknowledged. First, the absence of synchronized force plate data and electromyography (EMG) prevents the direct calculation of joint moments, mechanical power, and muscle activation patterns. For instance, Pfau et al. (31) demonstrated the value of combining synchronized kinetic and kinematic data to characterize loading during jump landing in agility dogs. Thus, our conclusions regarding “energy absorption” and the active versus passive cushioning dichotomy are derived from kinematic geometry and inferred neuromuscular synergies rather than direct kinetic or neuromuscular quantification. Future studies combining 3D kinematics with inverse dynamics and EMG are warranted to validate these proposed models. Second, while standard for intensive 3D marker-based animal studies, the sample size (*n* = 4) limits population-level generalizability and restricts the statistical power to detect subtle biomechanical differences. Consequently, the findings presented here should be considered hypothesis-generating rather than definitive, and caution must be exercised when extrapolating these results to the broader population of German Shepherds. However, despite this limited sample size, the data obtained from clinically healthy German Shepherds provide valuable preliminary kinematic information and serve as an important baseline scientific reference. These normative profiles of limb-specific movement patterns and functional differentiation are particularly useful for comparison with dogs presenting with musculoskeletal lesions, orthopedic disorders (such as hip dysplasia or CCL rupture), neurological conditions, or animals undergoing rehabilitation. Furthermore, the sample consisted exclusively of healthy German Shepherds; breed-specific conformational differences (e.g., angulation in sighthounds versus sturdier working breeds) may significantly alter the balance between passive and active cushioning strategies. Third, marker-based capture inherently involves skin artifact and restricts ecological validity, as evaluating dogs in uncontrolled, real-world agility environments is currently not feasible. To overcome these constraints, future veterinary sports medicine research should explore markerless, computer-vision-based kinematic profiling (43) and wearable sensor-based monitoring (leveraging advances in flexible and stretchable strain sensors) (44–46) integrated with machine learning to capture biomechanical patterns in real-world working environments (47–50). Recent reviews have underscored the growing role of both optical and sensor-based kinematic systems in veterinary practice, highlighting their potential for more accessible and ecologically valid gait assessment (51). Integrating such scalable field assessments with force plates and EMG is required to fully validate this dual-cushioning paradigm in true sporting environments. Ultimately, establishing this kinematic baseline has potential relevance for veterinary gait analysis, orthopedic evaluation, and rehabilitation research, providing a useful foundation for subsequent studies with larger sample populations and clinical case cohorts.

## 5 Conclusion

This study provides preliminary evidence suggesting that German Shepherds employ fundamentally distinct, limb-specific kinematic strategies for impact attenuation. The hindlimbs function as active, spring-like shock absorbers whose kinematics imply eccentric muscular control, which may predispose them to fatigue-related injuries such as CCL insufficiency. The forelimbs operate as passive, pendulum-like energy dissipators, relying on the extreme dorsiflexion of the carpus, which offers a mechanistic basis for understanding the high clinical incidence of carpal ligamentous injuries. While based on a limited sample and considered hypothesis-generating rather than definitive, these insights provide a crucial biomechanical framework for understanding canine orthopedic injury mechanisms and may serve as a normative baseline for evaluating pathological gait deviations in veterinary sports medicine and rehabilitation. Further studies with larger cohorts are necessary to confirm these findings.

## Data Availability

The original contributions presented in the study are included in the article/Supplementary material, further inquiries can be directed to the corresponding authors.
