# Supplementary material for: Comparative kinematic analysis of forelimb and hindlimb cushioning strategies in German Shepherd dogs: implications for injury prevention
Source: Front Vet Sci. 2026 Jul 7;13:1878605. doi: 10.3389/fvets.2026.1878605 (PMC13384870; doi:10.3389/fvets.2026.1878605)
Supplement: Supplementary file 1 [file Data_Sheet_1.PDF]

Supplementary Table S1. Individual dog-level kinematic observations and group means (Mean ± SD) during the cushioning phase across three gait conditions (walking, trotting, and drop-landing). Angular changes represent the range of motion, while vertical displacements represent the maximum downward movement relative to initial contact.

Variable	Gait	Dog A	Dog B	Dog C	Dog D	Group Mean ±
Carpal joint angular change (°)	Walking	28.9	24.9	30.2	28.3	28.1 ± 2.0
	Trotting	28.5	34	34.9	30.9	32.1 ± 2.5
	Drop-landing	41.1	41.7	43	41.9	41.9 ± 0.7
Shoulder joint angular change (°)	Walking	3.6	5.3	7.3	5.8	5.5 ± 1.3
	Trotting	4.4	6.9	5.6	4.7	5.4 ± 1.0
	Drop-landing	13.2	14.7	19.2	13.7	15.2 ± 2.4
Carpal vertical displacement (mm)	Walking	24.5	34	35.5	26.2	30.1 ± 4.8
	Trotting	31.3	33.6	34.4	31.3	32.7 ± 1.4
	Drop-landing	43.1	36.7	45	36	40.2 ± 3.9
Elbow vertical displacement (mm)	Walking	8.0	6.2	9.0	9.9	8.3 ± 1.4
	Trotting	17.8	18.6	20.7	15.3	18.1 ± 1.9
	Drop-landing	24.5	20.6	27.3	26	24.6 ± 2.5
Stifle joint angular change (°)	Walking	9.1	8.9	12.6	9.7	10.1 ± 1.5
	Trotting	14.4	13.7	15.3	13.8	14.3 ± 0.6
	Drop-landing	18.6	23.4	21.8	19.3	20.8 ± 1.9
Tarsal joint angular change (°)	Walking	2.2	1.4	2.9	2.4	2.2 ± 0.5
	Trotting	5.8	4.2	8.7	2.5	5.3 ± 2.3
	Drop-landing	5.4	4.2	7.3	5.6	5.6 ± 1.1
Stifle vertical displacement (mm)	Walking	13.6	11.3	13.9	12.3	12.8 ± 1.0
	Trotting	21.7	18.7	24.3	25.0	22.4 ± 2.5
	Drop-landing	31.7	35.4	37.2	32.8	34.3 ± 2.2
Tarsal vertical displacement (mm)	Walking	4.1	8.9	11.4	6.0	7.6 ± 2.8
	Trotting	9.5	6.9	8.1	6.6	7.8 ± 1.1
	Drop-landing	12.3	8.5	15.6	14.4	12.7 ± 2.7
